# The Social Nestwork: Tree Structure Determines Nest Placement in Kenyan Weaverbird Colonies

**DOI:** 10.1371/journal.pone.0088761

**Published:** 2014-02-13

**Authors:** Maria Angela Echeverry-Galvis, Jennifer K. Peterson, Rajmonda Sulo-Caceres

**Affiliations:** 1 Department of Ecology and Evolutionary Biology, Princeton University, Princeton, New Jersey, United States of America; 2 Department of Mathematics, Statistics, and Computer Science, University of Illinois at Chicago, Chicago, Illinois, United States of America; 3 Departamento de Ecologia y Territorio, Pontificia Universidad Javeriana, Bogotá Colombia; University of Regina, Canada

## Abstract

Group living is a life history strategy employed by many organisms. This strategy is often difficult to study because the exact boundaries of a group can be unclear. Weaverbirds present an ideal model for the study of group living, because their colonies occupy a space with discrete boundaries: a single tree. We examined one aspect of group living. nest placement, in three Kenyan weaverbird species: the Black-capped Weaver (*Pseudonigrita cabanisi*), Grey-capped Weaver (*P. arnaudi*) and White-browed Sparrow Weaver (*Ploceropasser mahali*). We asked which environmental, biological, and/or abiotic factors influenced their nest arrangement and location in a given tree. We used machine learning to analyze measurements taken from 16 trees and 516 nests outside the breeding season at the Mpala Research Station in Laikipia Kenya, along with climate data for the area. We found that tree architecture, number of nests per tree, and nest-specific characteristics were the main variables driving nest placement. Our results suggest that different Kenyan weaverbird species have similar priorities driving the selection of where a nest is placed within a given tree. Our work illustrates the advantage of using machine learning techniques to investigate biological questions.

## Introduction

As an evolutionary response to challenges of group living across different environments, group members display certain patterns of organization or behavior [1]–[4]. Individuals might adjust their location depending on environmental factors such as air temperature and wind [5]–[13], or social elements like territoriality and proximity to other species [1], [2], [14]–[18]. Distributions may also depend to some degree on the physical limitations of the group or when being studied, the scale of the study [3], [4], [19]. This variation in the placement of colony units may be due in part to the fact that group living confers various advantages and disadvantages to individual fitness [7], [9], [11]–[13], [20]. Possible advantages include access to mates and protection from predation due to more vigilance, while disadvantages likely include increased competition for resources and increased predation due to more salience. [15]–[18], [21]. Additionally, some colonies present cooperative breeding systems, in which reproductive fitness and relatedness may determine spatial arrangements of individuals [19]. Determining exactly which of the aforementioned factors drives the spatial arrangement of colony units is complex, from both an ecological and evolutionary perspective.

Colonies acting as information centers is one hypothesis as to why even seemingly disadvantageous aggregations are maintained in several species [20]. In a broad sense, they constitute centers where information is passively transferred with no preferential or structured distribution [21]. The formation of groups, and the chance to share information regarding abiotic conditions has been put forward as a factor influencing the evolution of social strategies such as cooperative breeding [1], [5].

It has been proposed that for the social weaverbirds of the Ploceidae and Passeridae families, abiotic and biotic conditions play key roles in determining individual nest location and nest architecture [22]–[25]. Some of the abiotic factors that determine nest placement within a colony include solar radiation, wind speed and precipitation [6], [26]–[30]. Additionally, competition could generate a repulsion area or distance between conspecifics [29]–[34], while predation risk could favor more aggregation and higher densities based on better predator visibility [14], [22], [24]. Some of these factors have been studied in collective behavior frameworks for different organisms such as insects, birds and eusocial mammals [26], [27], [29], [30], [35]. However, none of these ideas have been tested in colonies outside the breeding period, which is a longer period of time than the breeding period, and therefore could provide information about the fitness advantages of nest placement in the context of increased survival, independent of reproduction. Moreover, given that colony-forming species might use the same nest across both the breeding and non-breeding period throughout the year or for several years [31], [33], the study of nest location could reveal key variables that determine breeding success for the whole colony (in the case of cooperative breeders) or pair breeding success.

Nesting weaverbirds present a unique opportunity to study nest arrangement in a space with discrete edges, eliminating the question of where a colony's boundaries lie, a common problem in seabird colony research [14], [36]. Weaverbirds comprise around 108 species in 16 genera [35], [37], most of which tend to nest with conspecifics in a given tree [33], [38], and commonly have a separate roosting and breeding nest. This particular configuration of tightly arranged nest colonies with no sign of cooperative breeding has only been noted in birds from the Icterid family [36].

Our study aims to understand if the location of individual nests of the Black-capped Weaver (*Pseudonigrita cabanisi* Fischer & Reichenow 1884), Grey-capped Weaver (*P. arnaudi* Bonaparte 1850), and White-browed Sparrow Weaver (*Ploceropasser mahali* Smith 1983) within a tree (and therefore a colony), as well as their arrangement with respect to other nests have common patterns and/or governing parameters. Specifically, we asked if the location of a given nest is influenced by (1) tree structure, (2) environmental factors, or (3) its proximity to other nests.

## Methods

### Study site

Nest surveys were conducted in January 2010 at Mpala Research Center (MRC) in the Laikipia District of central Kenya (0°20′ N, 36°53′ E). Detailed weather information was obtained from local weather stations at MRC, including daily temperature, wind direction and wind speed for one month prior to and ending one month after the study.

**Figure 1:**
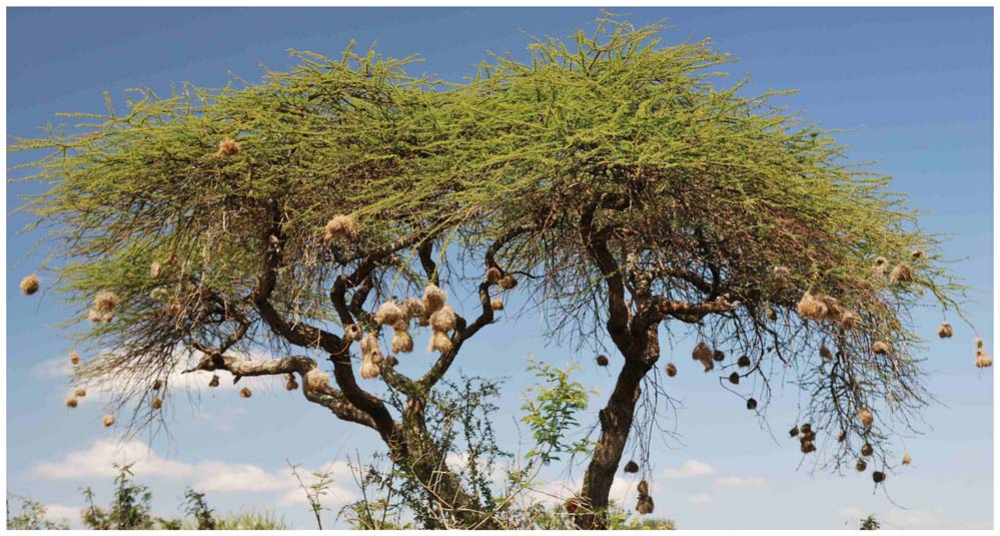
Tree 5. Tree located at Mpala Research Station with colonies of both Grey-capped and black-capped weavers.

We selected sixteen trees containing colonies of one or more of the three study species (Fig. 1). Each nest within a tree was recorded and the following measurements taken: (1) distance to the ground (using a telescopic graded bar); (2) horizontal distance to the trunk (measured linearly from the point of first branching in the main trunk); (3) entrance direction (facing downward toward the ground, east, north, west or south); (4) distance to closest neighbor, (categorized by increments of 10 cm, from 10–60 cm); (5) condition (“good,” when the nest was cohesive and in a rounded shape; “bad,” when the nest had gaping holes and/or was not securely attached to the branch; and “in construction,” when weaver birds were observed building it); and (6) whether it was actively in use by a weaverbird. Species ownership was determined either by direct observation or nest architecture [37]. Structural measurements for each tree were: (1) total canopy at the cross-section of the longest perpendicular canopy branches; (2) height at the highest point of the tree; (3) general branching pattern (dichotomous or multiple); and (4) diameter at breast height (DBH).

### Study species

Grey-capped Weavers (GC), *Pseudonigrita arnaudi* are gregarious birds that live in small, dense colonies [33] and are a common resident of African woodlands. Black-capped Weavers (BC), *Pseudonigrita cabanisi*, are ground-feeders that, similar to Grey-capped Weavers, are commonly found in grassy savannas below 1300 m. Both species are cooperative breeders, in which various individuals share nests within a tree [33], making a tree a coalition of groups of 2 to 8 individuals. White-browed Sparrow Weavers (WBSW), *Plocepasser mahali*, are a highly social species that live in small flocks that defend territories [24] and feed mainly on insects and occasionally seeds.

Field research was conducted under research permit authorized by Kenya Ministry of Education, Science and Technology number MOST 31/001/29C 80Vol.11 to D. I. Rubenstein and under authorization from Mpala Research Center. No animal was manipulated during the course of the fieldwork.

### Data Analysis

We used two main machine learning techniques: Principal Component Analysis (PCA) and Random Forests (RF) to identify the main predictors of nest arrangement in the trees.

PCA is a dimensionality reduction method that identifies the underlying factors explaining most of the variance in data [39]. We used the Hill Smith variant of linear PCA [40], since it can handle both quantitive and qualitative variables, as was the case of our dataset. To select the most important principal components, we considered the contribution criterion from Kaiser [41] and the interpretability criterion of Hatcher & Stepanski [42], in addition to percent variance explained by each component. Based on the Kaiser criterion, an important principal component should explain at least one original variable (the corresponding eigenvalue is at least one). The interepretability criteria, on the other hand, states that an important principal component is a linear combination of original variables all representing the same latent concept. We considered an original variable to be influential or important if its coefficient or weight in the principal component was at least 0.3 [41], [43].

We compared the linear PCA to the RF method, which offers an alternative analysis approach that avoids the linearity assumption made by the PCA method [44]. In addition, RF differs from PCA in that it computes the importance of original variables with respect to prediction, which in our case are those variables that best predict the local arrangment of the weaver nests. To represent such an arrangement, we used three different metrics as proxies of arrangement that provide information regarding the spacial distribution of the nests in a particular tree: (1) nest height relative to tree height; (2) nest height relative to the highest nest; and (3) the distance of the nest with respect to the farthest nest.

RF constructs an ensemble of decision trees (non-linear models) using bootstrap samples from the original values and outputs an average of their prediction results [44]. Within a tree, each node considers a randomized subset of the original variables. Bootstrapping, variable randomization and averaging are critical features that make RF a robust machine learning technique especially for analysis of complex, nonlinear and highly dimensional data [44]–[46]. RF computes two qualitative measures that describe the predictive power of the original measures: the Increased Mean Square Error (IncMSE) and Increased Impurity Index (IncNodePurity). IncMSE measures the effect on the predictive power when the value of a specific original variable is randomly permuted [44]. If the random permutation drastically changes the predicted value (as measured by the mean squared error), then the original variable is considered critical. IncNodePurity measures the total increase in the homogeneity of the data samples from splitting them on a given variable.

Spatial arrangement was also independently analyzed using visual exploration techniques to further understand how different measurements interacted and influenced the arrangements of the nests in the individual trees. Comparisons among variables in individual trees or nests were analyzed using ANOVA or Chi-square contingency tests when the data fit the normality assumptions; otherwise Wilcoxon signed-rank tests were used.

All analyses were performed in the statistical environment R, version 2.12.1 (R Development Core Team, 2010). PCA Hill-Smith was performed using the method “dudi.hillsmith”, which is part of the “ade4” library [47]. RF analysis was performed using the ‘randomForest’ library.

## Results

### Nest arrangement

We collected data for a total of 515 nests in 16 trees. Most of the colonies were found on *Acacia mellifera* trees (68.7%), followed by *A. xanthophloea* (25%) and by *A. etbaica* (6.25%). *A etbanica* was used exclusively by WBSW.

**Figure 2:**
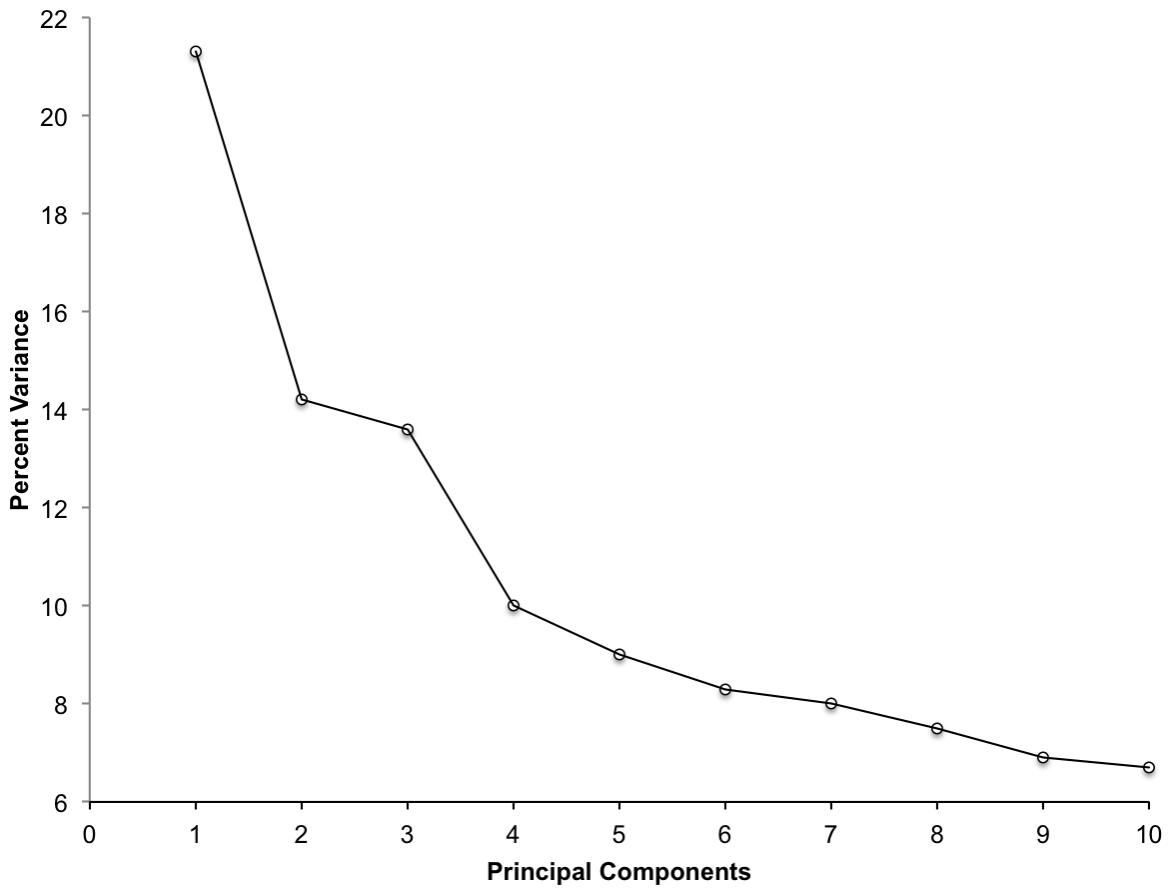
Screening plot from PCA Hill-Smith, showing corresponding variance of components. Total variance present in data equals 10 in the weaver-bird nest arrangements.

The scree plot (Fig. 2) for the Hill-Smith PCA method showed no clear-cut decrease in the variance explained by the top principal components. However, based on the Kaiser criterion, components 1 through 6 were of interest (ranging from 3.416–1.232, component 7 = 0.954 eigen-value). Components 4 through 6 either did not pass the interpretability criterion (with less than 3 influential variables) or contained influential variables that were not unique to them.

The first principal component explained 21.2% of the total variance (Table 1), and included canopy size, branching patterns, tree height and total number of nests in the tree. The second component explained 14.5% of the variance, for a cumulative total of 35.7%. There was no evidence from the PCA analysis that “species” played any determinant role in nest arrangement.

**Table 1:** Selected observed variables and corresponding coefficients based on interpretability and Kaiser criterion for PCA analyses of Weaver birds nest arrangement.

Component	Variable	Coefficient
1	Canopy size	0.77
	Branching pattern	0.68
	Tree height	0.63
	Total number of nests	0.42
2	Condition	0.75
	Use	0.62
	Entrance orientation	0.59
3	Tree species	0.73
	Entrance orientation	0.41

**Figure 3:**
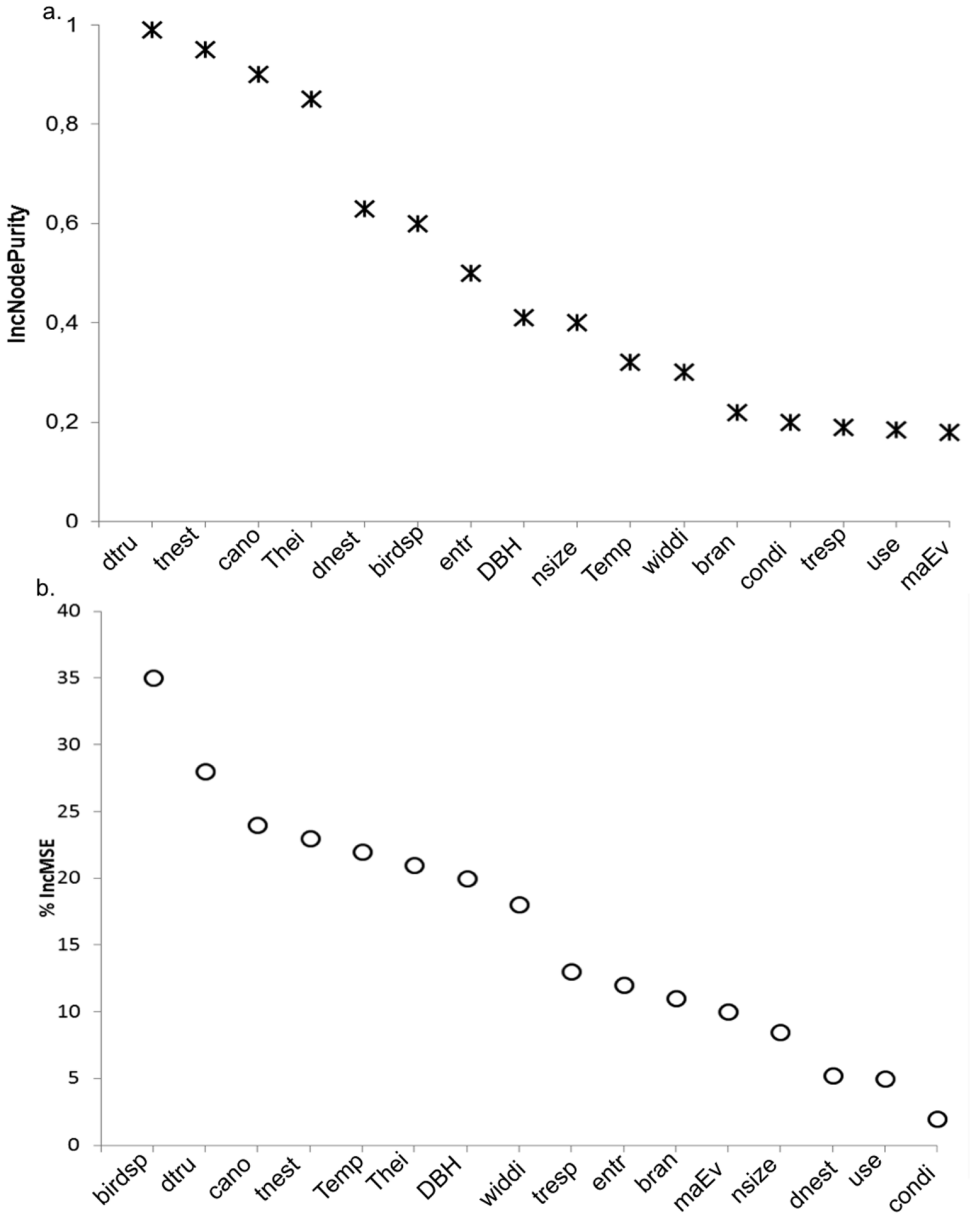
Random Forest evaluation. Mean square-error (%IncMSE) and node purity (IncNodePurity) corresponding to the original variables when nest location is represented by nest height over tree height. dtru: distance to the trunk, tnest: total number of nests, cano: canopy size, Thei: tree height, dnest: distance to closes neighbour, birdsp: bird species, entr: entrance, DBH: diameter at breast height, nsize: nest size, Temp: temperature, widdi: wind direction, bran: branching pattern, condi: condition of the nest, tresp: tree species, use: whether in use or not, masEv: if the nest was part of a mass event.

The best predictors for nest arrangement in each of the tree RF models were total number of nests and canopy size (Table 2). Model 3 (nest location represented by normalized nest height relative to tree height) captured most of the variance in the data based on both IncMSE and IncNodePurity (Fig. 3a and b). RF analysis identified total number of nests, canopy size, distance to the trunk, and bird species as important original variables.

**Table 2:** Random Forest models using three normalized variables as representations of nest location for weaver birds in Mpala Research Station.

Predicted variable	R^2^ error	Variance explained	Important predictor variables
(1) Normalized distance from trunk (with respect to farthest nest. A horizontal perspective of nest distribution).	0.0179	47.86	Tree height, nests' height, total number of nests, canopy size. Total (4).
(2) Normalized nest height (with respect to highest nest. A vertical perspective in relation to other nests).	0.0131	44.24	Total number of nests, distance from the trunk, distance between nests, bird species, temperature, canopy size. Total (6)
(3) Normalized nest height (with respect to tree height. Also a vertical perspective but in relation to available space).	0.008	53.68	Total number of nests, canopy size, distance from the trunk, bird species. Total (4)

Based on these models, we plotted nest measurement data to visually explore their spatial distribution. We chose canopy, total number of nests and distance from the trunk (as dictated by PCA and RF analysis). We also overlaid bird species information and tree species information on these plots to further identify any potential patterns.

### Individual tree analysis

GC and BC segregated frequently in relation to canopy width, total number of nests and distance from the trunk. However, GC was recorded nesting on 85.7% of the trees with BC, while WBSW nest independently, constructed fewer nests that were close together and used trees with smaller canopies. This species was also exclusively found in *A. etbanica*, where nests clustered based on the aforementioned variables.

**Figure 4:**
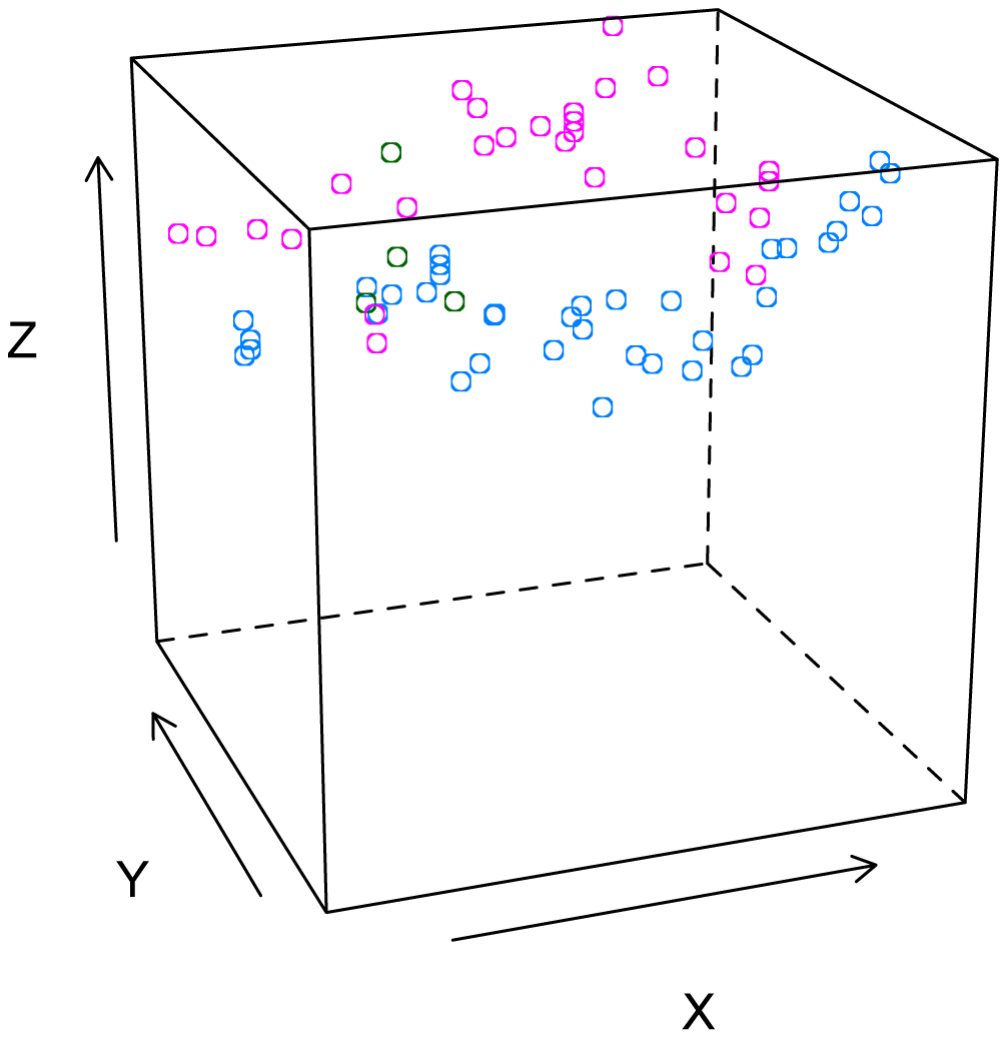
Tree 12. Schematic plot of nests on tree 12 where both Black-capped and Grey-capped weavers roosted. Axis x, y and z represent the nest position on the tree. Blue circles: Black-capped Weaver, pink circles: Grey-capped Weaver, green circles: species not determined due to poor nest condition.

In four out of the five trees where BC and GC were present, we found different species clusters to one side of the tree. GC was found in the northwestern portion of the tree (χ^2^_7_: 1.6, p = 0.02) or in lower branches (χ^2^_2_: 7.0, p = 0.03) while BC was located in the upper branches and spread out across the tree. For example, in a plot representation of tree 12, coded by bird species (Fig. 4), it is clear that both species cluster to different parts of the available tree space.

For all the nests surveyed, we found 66 occasions where nests (ranging from 2–4), were physically in contact in a so-called “mass event”. There was a strong correlation between number of mass events and total number of nests per tree (Pearson r^2^ = 0.82, p<0.0001), but no correlation between the total area of the canopy or the tree height (p≤0.12). Of the mass events in trees with both BC and GC, 12% corresponded to heterospecific situations, with no case involving the three study species. In all trees in which the mass events were observed, the participating nests were in good condition (87% N = 152) with downward-facing entrances (75%).

BC nest size did not differ when they nested on their own or with GC, (Wilcoxon signed-rank test: N = 178, z = −1.45, p = 0.15), while GC built significantly bigger nests when nesting on their own (Wilcoxon signed-rank test: N = 230, z = 3.60, p = 0.0003).

No significant differences were found between nest condition or the likelihood of a nest being used, and any other physical parameter of the tree (condition/use vs. wind position and condition/use vs. relative height per tree (ANOVAs p≥0.24, N = 428), and only WSBW showed a clustered distribution to the leeward portion of the trees in which they nested (χ^2^_1_ = 4.02, p = 0.041, N = 28).

## Discussion

Similar to what has been found in marine colony breeders, the main factors determining where a nest would be located in an existing colony, seems to be highly correlated with physically-available space within an previously-selected tree [14], [17], [18], [48]. The agreement in explanatory variables between marine bird colonies and weaverbird colonies points to common limitations and trade-offs that determine the location of individuals within the colonies. However, the lack of any clear-cut differences in the variance explained by PCA principal components and the lack of consistency between the outputs of the PCA and RF analyses prompt us to suggest that more complex interactions likely dictate nest position.

The RF model showed bird species as an important variable in predicting nest height relative to the highest nest in the tree, while PCA analysis did not. This could be due to strong similarities in nest position between the Black-capped and Grey-capped weavers, which constitute the majority of the data. If this is the case, then RF should perform better than PCA with under-represented species such as the White-browed Sparrow Weaver in our dataset. The role of branching pattern also differs between analyses, with PCA picking it up as important. It is possible that a more complex branching pattern could generate a bigger fractal space in which nests could be placed [3], however given the size of the nests and the physical limitation of space per tree, this is highly unlikely in the case of weaverbirds. From an analytical, computational point of view, we consider RF a more appropriate technique when dealing with multiple variables at different levels since it can handle non-linear relationships and has a better performance in terms of the variance explained [44], [49]–[51]. We suggest RF as a better methodological/analytical tool to explain complex relationships regarding ecological and physical interactions in comparison to more traditional methods [46], [52].

RF was able to predict nest location based on a smaller number of variables, including two that were not detected by the PCA analysis: distance from the trunk and bird species. Distance from the trunk could be relevant as a possible mechanism for predation deterrence, as nests located further away from the main trunk, on smaller, thinner branches would be more difficult for most predators (such as hawks, eagles and some snakes) to reach [35]. Only WSBS consistently placed their nests just in leeward areas of the trees, as with the breeding nests [6], [38], suggesting that species response to weather parameters might be more species-specific than previously found [53], or that BC and GC do not show arrangement constraints based on such parameters. The weather data we used was at a coarse resolution and more detailed observations of temperature and wind at nests could lead to a different conclusion.

Canopy size and tree height (referring to tree architecture and space availability), are the principle variables influencing nest location in the trees, in accordance with our initial expectations. These factors with the possible addition of branching pattern suggest that the tree's geometry [54] is the biggest constraint when choosing specific locations within the colony to build a dormitory; however, this can also be related to nest success during the breeding season [53]. In their extensive study of nest architecture, Collias and Collias [55] suggested that larger numbers of GC nests would be found in trees >6 m tall, which is consistent with our observations. Trees with such characteristic in our dataset also supported more BC nests and more nests of both species in instances where they shared a single tree.

Collias and Collias [38] reported only one tree in which GC nests were found in physical contact forming “mass events.” Craig [56] reported that family groups of this species could fuse nests together into masses of up to 20 nests during the breeding period, however these mass events have never been reported between different species. Heterospecificity in nest contact is not in line with the idea that such aggregations would have been at the origin of massive nesting processes such as those found in the Sociable weaver (*Philetairus socius*) [36], [55]. The final explanation of the possible evolution and ancestral character of mass events in nest arrangements awaits two main inputs, (1) a complete and detailed phylogenetic study of the families in the weavers groups, and (2) more information on the frequency of these mass events between more than two species. It should be noted that, even when there was a positive correlation between number of mass events and total number of nests, no such relation was found between the number of mass events and canopy area or tree height, contrary to previous assumptions [55].

GC constructed bigger nests when alone in a tree than when sharing it with BC, which could indicate a trade-off between sharing a tree and nest size, perhaps due to both species using similar nesting material [37]. This lends credence to the hypothesis that a main selection pressure for nest arrangement and architecture is interspecific competition for nest material [57], [58], such that nest size is more affected by interspecific rather than intraspecific competition. Nests are built mainly for breeding purposes, and are usually constructed just prior to the breeding season, although in long term colonies like those of weavers and Icterid South American birds, nests may be constructed *de-novo* even outside the breeding period (M. A. Echeverry-Galvis *pers. obser*.). Additionally, nest condition and size as determined in this study, could also be regulated by a social hierarchy among the cooperative breeders. Therefore, it remains to be tested whether nest size differences are sustained into the breeding season and if they are indeed possible proxies of social status within a colony.

Overall, we found that weaverbirds in Kenya select nest locations according to the space available within their tree, with canopy size being the best predictor of the arrangement for the Black-capped and Grey-capped weavers. We demonstrate that machine learning techniques such as Random Forest outperform more conventional analytical tools such as linear PCA, thanks to their ability to model complex nonlinear relations and their robustness to noise. Applying these techniques to the study of other colonial-living organisms might better enable researchers to identify new ecological and evolutionary factors at play in the spatial arrangements of group living organisms.
